# Infrared inhibition of embryonic hearts

**DOI:** 10.1117/1.JBO.21.6.060505

**Published:** 2016-06-27

**Authors:** Yves T. Wang, Andrew M. Rollins, Michael W. Jenkins

**Affiliations:** a Case Western Reserve University, Department of Pediatrics, 10900 Euclid Avenue, Cleveland, Ohio 44106, United States; b Case Western Reserve University, Department of Biomedical Engineering, 10900 Euclid Avenue, Cleveland, Ohio 44106, United States

**Keywords:** infrared inhibition, infrared control, cardiac electrophysiology, lasers

## Abstract

Infrared control is a new technique that uses pulsed infrared lasers to thermally alter electrical activity. Originally developed for nerves, we have applied this technology to embryonic hearts using a quail model, previously demonstrating infrared stimulation and, here, infrared inhibition. Infrared inhibition enables repeatable and reversible block, stopping cardiac contractions for several seconds. Normal beating resumes after the laser is turned off. The block can be spatially specific, affecting propagation on the ventricle or initiation on the atrium. Optical mapping showed that the block affects action potentials and not just calcium or contraction. Increased resting intracellular calcium was observed after a 30-s exposure to the inhibition laser, which likely resulted in reduced mechanical function. Further optimization of the laser illumination should reduce potential damage. Stopping cardiac contractions by disrupting electrical activity with infrared inhibition has the potential to be a powerful tool for studying the developing heart.

Infrared control is a powerful new technology for controlling excitable cells, enabling new studies in small cardiac samples. It has several advantages over conventional electrical techniques, including being contact-free and having higher spatial precision. This is critical for studies of cardiac development in tiny embryonic hearts as electrical techniques interfere with electrical recordings, are prone to cause damage, and may be difficult to position.^1^ Also, unlike optogenetics, infrared control does not require genetic manipulation, making it easier to implement and suitable in a wide variety of models.

Infrared control was first developed as infrared neural stimulation and uses pulsed infrared laser light to elicit firing of an action potential^2^ through a thermal effect,^3^ where the rapid temperature changes affect membrane capacitance^4^ and mitochondrial calcium cycling.^5^^,^^6^ It has since been used in a wide variety of animal models and tissues.^2^^,^^7^^–^^15^ We have previously demonstrated that infrared stimulation can be used to pace embryonic^1^^,^^16^ and adult hearts.^17^ Infrared stimulation has also been demonstrated in isolated cardiomyocytes.^5^

We have recently shown that infrared control can also be used to inhibit electrical activity.^14^ Infrared inhibition was achieved using the same infrared laser as used for infrared stimulation, but with different pulse parameters, and also works through a thermal effect where infrared light is absorbed by water and converted to heat. We have shown that the activation and propagation of action potentials can be stopped,^14^^,^^15^ and that the onset response from kilohertz high-frequency alternating current nerve block can be prevented.^18^ Inhibition could be achieved rapidly with high spatial selectivity based on laser spot positioning.^14^ The mechanism is hypothesized to be a heat block,^14^ a localized version of inhibition through globally increased temperature.^19^^,^^20^ While there are several other methods of blocking the propagation of action potentials—including pharmacological agents,^21^ applied current,^22^^,^^23^ and cooling^24^—they lack the rapidity and spatial specificity of infrared inhibition, with the exception of optogenetics,^25^ which is limited by the requirement of genetic manipulation.

Here, we demonstrate reversible infrared inhibition of early embryonic avian hearts, both in intact embryos and excised hearts, with spatial selectivity, and we further investigate the target of inhibition and potential damage.

Quail eggs (*Coturnix coturnix communis*; Boyd’s Bird Company, Pullman, Washington) were incubated for 2 days to Hamburger–Hamilton^26^ stage 14 in a humidified, forced draft incubator (G.Q.F Manufacturing, Savannah, Georgia) at 38°C. At this stage of development, the tubular heart is looped and has begun to beat. All animal husbandry and experiments were conducted in compliance with the National Institutes of Health’s Guide for the Care and Use of Laboratory Animals with the approval of the Institutional Animal Care and Use Committee at Case Western Reserve University.

Imaging was performed with a Zeiss Axio Scope.A1 microscope with a 5× objective and a 0.33× magnification tube lens (Carl Zeiss Microscopy, Thornwood, New York). Illumination for brightfield imaging was provided by ambient light, while a solid-state white-light source (Lumincor, Beaverton, Oregon) was used for fluorescence imaging. For inhibition, a 15-W PUMA laser (1463-nm, QPhotonics, Ann Arbor, Michigan) was coupled into a 600-μm diameter multimode fiber ending with a collimating lens (Ocean Optics, Dunedin, Florida) creating a measured full-width half-max spot size of ∼800  μm and illuminated the sample from below the chamber. The laser was pulsed at 200-Hz with 200-μs pulse widths, as used previously with nerves,^14^ at 0.33  mJ/pulse (1.65-W peak power), producing a calculated per-pulse radiant exposure of $66\;\mathrm{mJ}/{\mathrm{cm}}^{2}$.

To demonstrate the feasibility of using infrared inhibition to stop the beating of the heart in an early embryo, an intact embryo was removed from the yolk and placed in a glass-bottomed imaging chamber in 1 mL of Tyrode’s solution. Brightfield images were recorded of the embryo’s heart before, during, and after inhibition (Fig. 1 and Videos 1–3). The intensity of an arbitrary pixel on the edge of the heart (traces) reflects the motion of the heart as the tissue moves, indicating the heart rate.^1^ The heartbeat was inhibited three times with 18 to 25 s of exposure to the inhibition laser, with normal beating resuming after the laser was turned off.

**Fig. 1:**
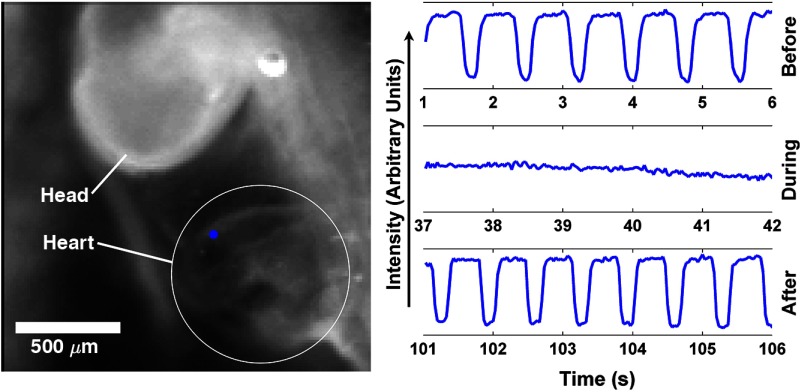
Demonstration of infrared inhibition in an intact embryo. Traces of pixel intensity as a surrogate for cardiac contractions at the blue dot on the ventricle are shown for before, during, and after infrared inhibition. Supplemental movies show the cardiac movement before (Video 1), during (Video 2), and after (Video 3) inhibition. (Video 1, MPEG, 0.2 MB [URL: http://dx.doi.org/10.1117/1.JBO.21.6.060505.1]; Video 2, MPEG, 0.1 MB [URL: http://dx.doi.org/10.1117/1.JBO.21.6.060505.2]; Video 3, MPEG, 0.2 MB [URL: http://dx.doi.org/10.1117/1.JBO.21.6.060505.3]).

Next, to investigate the spatial selectivity of the inhibition, hearts were excised into the imaging chamber with 1 mL of Tyrode’s solution. Excising the heart enabled more precise positioning of the inhibition laser on the heart and also allowed the target tissue to be placed in contact with the glass bottom, minimizing the amount of water that the inhibition laser passed through before reaching the tissue. In total, inhibition was successfully achieved in all 18 experimental embryos, with one to three repetitions per embryo, and recovery of normal beating occurred in all but one heart. Inhibition in excised hearts was established within 1 to 15 s of laser exposure and normal beating resumed within 40 s after the end of laser exposure, with a single exception, which experienced arrhythmias during recovery and required 75 s before normal rhythm was restored.

Positioning the inhibition laser on the ventricle resulted in blocking propagation of contraction at that area of the ventricle, resulting in the abolition of motion in both the ventricle and in the outflow tract [Fig. 2(a)]. By contrast, positioning the inhibition laser on the atrium resulted in blocking the initiation of contraction, resulting in the abolition of motion in the entire heart [Fig. 2(b)].

**Fig. 2:**
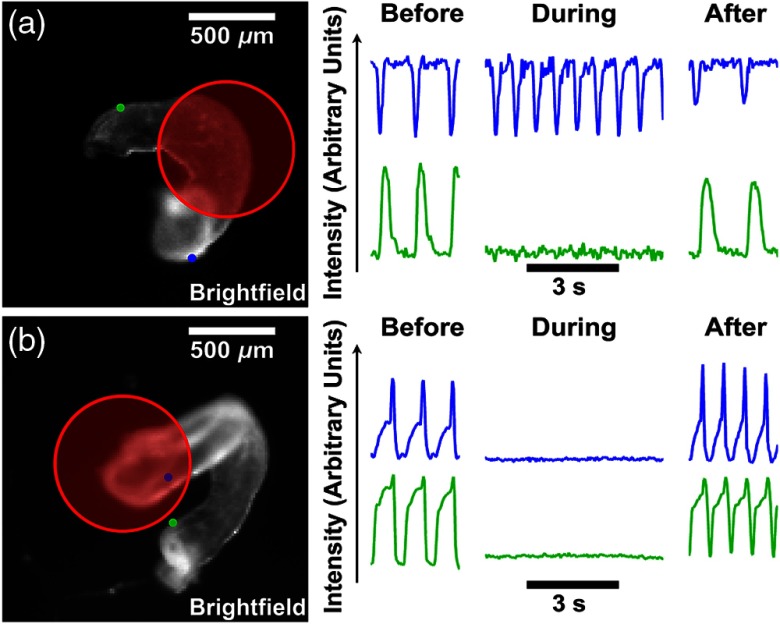
Spatial selectivity of infrared inhibition. (a) When the inhibition laser (red circle) was positioned on the ventricle, contractions were stopped in the outflow tract (green dot and lower traces) but not in the atrium (blue dot and upper traces). (b) When the inhibition laser was positioned on the atrium, all contractions were stopped. In both cases, normal contractions resumed after the inhibition laser was turned off.

Optical mapping of intracellular calcium and transmembrane potential was then used to explore the target of inhibition. Normal contractions in the heart are initially triggered by an action potential that depolarizes the cardiomyocyte cell membrane. This results in an influx of extracellular calcium through voltage-gated calcium channels that induces the release of calcium from the sarcoplasmic reticulum, which then triggers the movement of myosin that causes the contraction. To image intracellular calcium transients, excised hearts were stained with Fluo-4 in the presence of a nonionic surfactant Pluronic F-127 (Life Technologies, Carlsbad, California). No excitation–contraction uncoupler was used to abolish motion, so recordings show motion artifacts, but the presence or absence of calcium transients remains clear. Hearts were imaged before, during, and after inhibition on the ventricle [Fig. 3(a)]. Calcium transients were abolished at the site of the inhibition and downstream along the heart tube without affecting those upstream in the atrium and resumed after the inhibition laser was turned off.

**Fig. 3:**
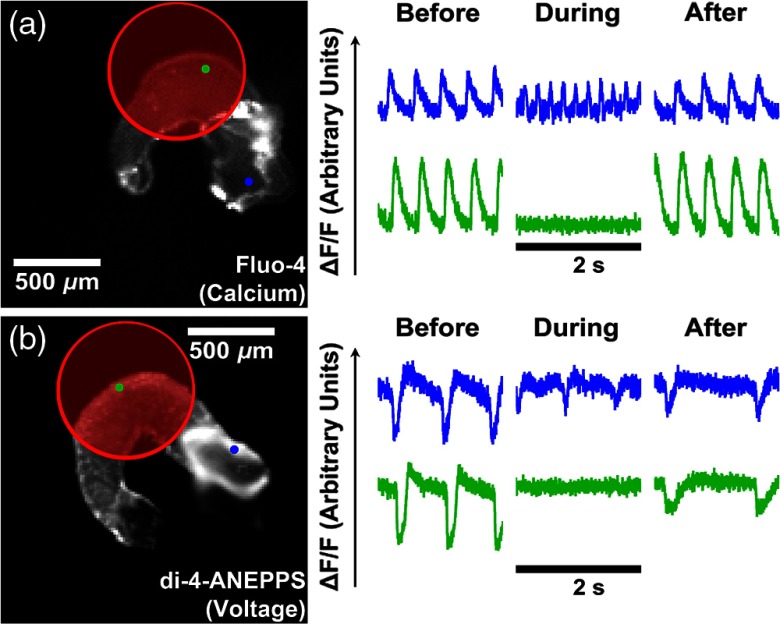
Optical mapping during infrared inhibition. (a) Calcium and (b) voltage optical mapping were taken before, during, and after inhibition with the laser positioned on the ventricle (red circle). Infrared inhibition abolished calcium transients and membrane depolarization in the ventricle (green dot and lower traces) but not the atrium (blue dot and upper traces). An excitation–contraction uncoupler was used for voltage only, which likely contributed to the poor health of the heart after inhibition.

Optical recordings of membrane voltage were acquired from excised hearts stained with di-4-ANEPPS. Hearts were imaged before, during, and after inhibition on the ventricle [Fig. 3(b)]. Membrane depolarization stopped both at the site of inhibition and further downstream of normal conduction on the outflow tract. Meanwhile, upstream in the atrium, membrane depolarization continued. Action potentials resumed in the ventricle after the inhibition laser was turned off. Due to the nature of the signal produced by the dye, cytochalasin D was used to abolish movement, as motion artifacts would have completely obscured the recordings. The exposure to this drug caused additional stress, resulting in a slowed heart rate during and after inhibition, as opposed to the expected increased heart rate due to a moderate increase in temperature as observed without cytochalasin D [Figs. 2(a) and 3(a)]. These optical mapping results show that infrared inhibition blocks the electrical activity (membrane depolarization) rather than just calcium flux or mechanical motion.

Both infrared stimulation and inhibition work through the mechanism of the infrared laser light being absorbed by water, which converts the energy into heat.^3^^,^^14^ While numerous studies have shown that infrared stimulation can be achieved without thermal damage,^1^^–^^4^^,^^9^^–^^17^ the infrared inhibition demonstrated in this study deposits a greater amount of energy into the tissue. To determine the change in temperature induced by the infrared inhibition, we used a thermal camera with ResearchIR software (FLIR A325sc, Wilsonville, Oregon) to image the temperature of the cross-section parallel to the laser beam in a water phantom. The maximum steady-state temperature increase was overlaid over a representation of a heart cross-section based on the spatial positioning of the center of inhibition laser relative to the excised embryonic hearts in this study. This analysis showed that the temperature increase experienced by the tissue of the 3-D tubular heart ranged between 10°C and 15°C, a great enough increase such that thermal damage is a concern.

To test for thermally induced necrosis, hearts were stained with propidium iodide, a membrane impermeant dye, for 10 min after 30 s of exposure to the inhibition laser. No increase in staining was detected, indicating that infrared inhibition did not result in compromised cell membrane via necrosis.

However, using Fluo-4 staining, we did detect a large increase in calcium concentration during the resting phase of the action potential (Fig. 4) on the side of the heart proximal to the laser, where the temperature increase was the greatest, as well as a smaller increase on the distal side. The increased intracellular calcium likely reduces cardiac contractility.^27^ Significant increases in intracellular calcium can also trigger apoptosis,^28^ but would not be detectable by assays for several hours and it is unlikely that a sufficiently large calcium spike was produced in these experiments.

**Fig. 4:**
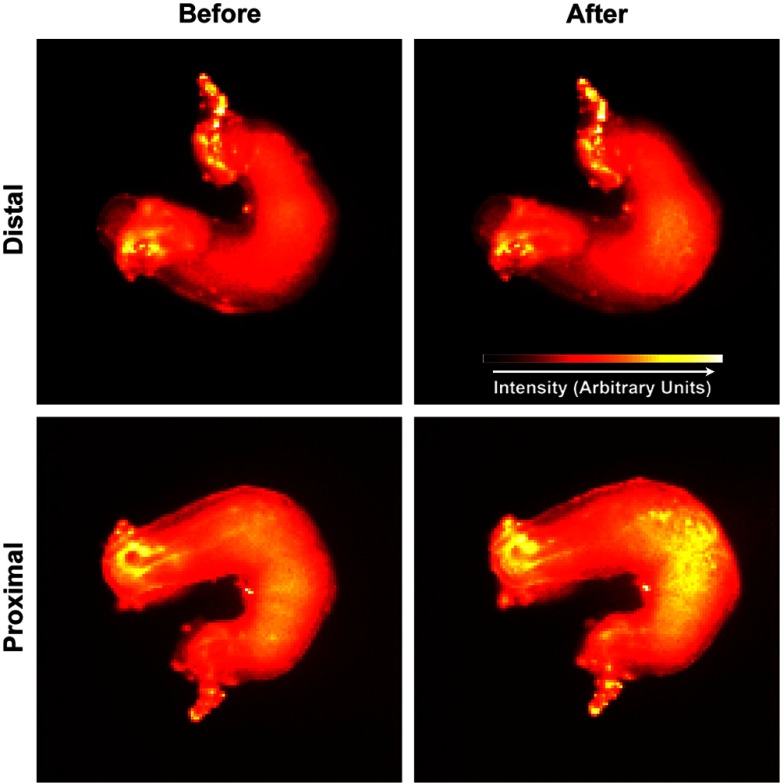
Resting intracellular calcium levels. Images were taken before and after a 30-s exposure to the inhibition laser on both the distal and proximal sides of the heart from the laser. A significant increase in intracellular calcium can be seen on the proximal side on the ventricle, where the laser was positioned. A small increase can also be seen on the distal side on the ventricle.

The damage tests were done with a longer single exposure (30 s) than used for any of the other experiments included in this study (typically 10 to 15 s with a maximum of 25 s). Since some hearts were inhibited up to three times without any detectable effect on mechanical function, it is possible that shorter exposures avoid damage. Additional studies will be conducted to determine the thresholds for mechanical dysfunction and apoptosis after optimization of the inhibition laser parameters.

To reduce possible damage to the heart, the power of the inhibition laser should be minimized. In this study, the power was not optimized to avoid damage. Rather, the power was selected to provide a high success rate on the first attempt, so in many cases, the laser power could be decreased to reduce potential damage.

Additionally, in the tubular embryonic heart, it is necessary to inhibit conduction of an entire cross-section; otherwise, action potentials would continue to propagate through any gap in the block. Changing the illumination profile on the heart could also reduce potential damage by avoiding over illumination of tissue proximal to the laser in an effort to reach the block threshold distally. Radiant exposure on the proximal side of the heart could be reduced by use of multiple illumination points so that the light would not have to penetrate across the entire heart tube. Radiant exposure in the center of the illumination spot could also be reduced by reshaping the beam to a more uniform profile to deliver energy more evenly to the entire illumination spot, rather than the Gaussian beam profile used here, where the majority of the delivered energy is concentrated at the center of the illumination spot. This would also likely allow inhibition with a smaller spot size, reducing the chance of off-target effects from heating other tissues.

Furthermore, the temporal profile of the laser could be altered to reduce damage. It may be desirable to use a variable-power illumination protocol with a higher initial and lower steady-state laser powers. Having a higher initial power would allow the temperature to more quickly rise to the block threshold, reducing the exposure time before inhibition is established. Also, a lower steady-state temperature may be sufficient to maintain block after inhibition has already been established, reducing the long-term radiant exposure.

In conclusion, we demonstrate here that infrared light can be used to inhibit cardiac activity in early embryos. This inhibition can be achieved with spatial selectivity, stopping the whole heart by targeting the atrium or part of the heart by targeting the ventricle. With optical mapping, we show that infrared inhibition stops contractions by inhibiting action potentials, which results in the abolition of intracellular calcium transients and contraction. Increased resting intracellular calcium was detected, which may indicate temporary mechanical attenuation at low levels or potential tissue damage at high levels, but short exposures or further optimization of the laser parameters will likely decrease any chances of damage during inhibition. Infrared inhibition has many potential applications in the embryonic heart, including enabling cardiac imaging without gating, allowing injections or tissue collection in a temporarily still heart, and producing disease models by disrupting normal function. Further in the future, the ability to inhibit cardiac electrical activity with high spatial specificity may enable novel experimental studies or more targeted treatments of arrhythmias.
